# Hypercalcaemia with disseminated osteolytic lesions: a rare presentation of childhood acute lymphoblastic leukaemia

**DOI:** 10.3332/ecancer.2015.542

**Published:** 2015-05-26

**Authors:** Rajitha Lokadasan, Shruti Prem, Sumod Mathew Koshy, A V Jayasudha

**Affiliations:** 1 Department of Medical Oncology, Regional Cancer Centre, Trivandrum, Kerala 695011, India; 2 Department of Imageology, Regional Cancer Centre, Trivandrum, Kerala 695011, India; 3 Department of Pathology, Regional Cancer Centre, Trivandrum, Kerala 695011, India

**Keywords:** acute lymphoblastic leukaemia, hypercalcaemia, osteolytic bone lesions

## Abstract

Acute lymphoblastic leukaemia (ALL) presenting with hypercalcaemia and lytic bone lesions is a rare event in children unlike adults. We report a 15-year-old boy with acute lymphoblastic leukaemia and hypercalcaemia. He had normal peripheral blood count and the peripheral smear did not show blast. The bone marrow examination revealed Pre B ALL phenotype with aberrant expression of CD13. The skeletal survey showed osteolytic lesions. Hypercalcaemia was treated with zoledronic acid. He attained remission only after three lines of intensive chemotherapy protocols. He was planned for stem cell transplant. Meanwhile, he relapsed and died. A review of the literature also highlights characteristics similar to our case.

## Introduction

Acute lymphoblastic leukaemia (ALL) is one of the commonest malignancies in children and adolescents. It represents approximately 25% of cancers diagnosed in children of less than 15 years of age [1]. ALL characteristically presents with fever, arthralgia, lymphadenopathy, and bleeding manifestations. WHO classifies ALL immunophenotypically to B and T ALL. Certain recurrent chromosomal abnormalities are correlated with prognosis in B ALL [2]. Chromosomal abnormalities, such as high hyperdiploidy t(12, 21), are associated with favourable prognosis. Other, like t(9, 22), mixed-lineage leukaemia (MLL) rearrangements have been shown to have poor prognosis. In the current protocol, ALL with unfavourable cytogenetics is treated with intensive chemotherapy followed by stem cell transplant. Among children with ALL, 95% attain remission, and approximately, 80% of them are expected to be long-term survivors [3].

Only a few cases of B ALL have been reported that presented with hypercalcaemia and generalised bone lesions [4]. Children with ALL who present with hypercalcaemia have been reported to have a distinctive clinical profile, pathological features, biology, and poor response to treatment. Here, we report a 15-year-old boy with ALL who presented with disseminated bone lesions and impending pathological fracture along with hypercalcaemia. He had a chemorefractory disease and attained remission only after the third line of intensive chemotherapy. However, while on chemotherapy, he relapsed and died.

This case report aims to identify the subset of patients with ALL who are unlikely to respond to conventional chemotherapy. This may help in anticipating clinical problems and also in selecting those patients for appropriate upfront intensive chemotherapy and stem cell transplant.

## Case Report

A 15-year-old boy presented with low-grade fever and severe bone pain of two months duration. He had anorexia and polyuria for the previous two weeks. Two weeks prior to admission, he had severe pain in the right hip joint and was unable to walk. System examination showed generalised lymphadenopathy and hepatosplenomegaly.

Laboratory investigations revealed a haemoglobin of 6.2 g/dl, WBC count of 2,100/mm3, and platelet count of 23,000/mm3. Peripheral smear showed occasional circulating blasts. The baseline serum creatinine was 1.1 mg/dl which rose to 2.3 mg/dl over the next few days, serum uric acid was 9.6 mg/dl, and serum calcium was 17.2 mg/dl. Serum phosphorous levels and liver function tests were normal, and serum LDH was 1,334 u/ml.

### Radiology

An X-ray of the pelvis showed multiple permeative lesions involving the pelvic bones and the visualised proximal femurs including the neck. There is periosteal reaction and foreshortening of the neck of left femur. An X-ray of the knee joint showed multiple lytic in the bones around the knee joint bilaterally in the metaphyseal region.

Computed tomography (CT) of the pelvis showed multiple lytic lesions involving the sacrum, bilateral iliac bones, acetabulum, head, neck, and the proximal shaft of both femurs with endosteal scalloping and cortical breaks suggestive of leukaemic infiltrates. Linear periosteal reaction could be visualised in bilateral metadiaphyseal region of femur.

### Pathology

Bone marrow aspiration revealed 59% blasts that were positive for CD10, 19, 20, 79a, and HLA DR, with aberrant expression of myeloid markers CD13 and 33. Cytogenetics by conventional karyotyping was normal and RT-PCR for bcr-abl was negative. Cerebrospinal fluid (CSF) cytology was negative for malignant cells.

### Treatment

He was initially treated with saline diuresis, frusemide, steroids, and i.v. calcitonin for the first three days to ameliorate the severe hypercalcaemia; however, his calcium continued to be persistently elevated and serum creatinine showed rising trend. He then received single dose of injection zoledronic acid 4 mg i.v. and was also started on ALL induction chemotherapy with BFM-95 protocol. He also received 8Gy single-dose radiotherapy (RT) to the right neck of the femur for impending pathological fracture and to relieve pain.

Twenty-four hours after bisphosphonate therapy, he showed progressive decline in serum calcium levels and rapid normalisation of serum creatinine. He developed severe hypocalcaemia and hypomagnesaemia over the next one week despite i.v. supplementation of calcium and magnesium and had an episode of generalised tonic clonic seizures secondary to the severe metabolic derangement. He continued to require high doses of intravenous calcium and magnesium for the next three weeks.

He received four drug induction chemotherapy with BFM protocol. This included prednisolone (60 mg/m^2^/day for 28 days), daunorubicin (25 mg/m^2^/day weekly for 4 doses), vincristine (1.5 mg/weekly for 4 weekly doses), and peg asparaginase (2,500 IU/m^2^/dose every 3 days for 8 doses starting from day 3). After completion of 4 weeks of induction chemotherapy, his blood counts normalised, but the bone marrow showed 26% blasts. He then received re-induction with BFM high-risk protocol, but even at the end of the second induction, his bone marrow showed persistent disease. Subsequently, he received a hyperfractionated chemotherapy regimen with alternate courses of 2 combinations given for eight cycles. Course B consists of cyclophosphamide, vincristine, doxorubicin and dexamethasone. Course B consists of methotrexate and cytarabine. Considering the aggressiveness of the disease and since he had a human leukocyte antigen (HLA)-matched sister, it was planned to consolidate him with an allogeneic bone marrow transplant. However, within a short span of less than one month, he relapsed and died.

## Discussion

Hypercalcaemia (defined as a serum calcium of more than 12 mg/dl) in association with osteolytic bone lesions is a common presenting feature in some adult haematological malignancies such as myeloma and adult T cell lymphoma/leukaemia. However, it is rarely seen at presentation in paediatric cancers. In a large retrospective study [4] of more than 6,000 paediatric cancer patients from St. Jude’s children’s cancer hospital over a 29-year period, only 0.4% had hypercalcaemia during the course of malignancy. In the subgroup of patients with acute leukaemia/lymphoma (2,816 patients), the incidence of hypercalcaemia at diagnosis was less than 0.3%. Another series by Hibi *et al* [5], reported a higher incidence (4.8%) in 83 patients with pre-B ALL. The commonest paediatric malignancies associated with hypercalcaemia are rhabdomyosarcoma, hepatoblastoma, lymphoma, brain tumours, neuroblastoma, angiosarcoma, and less commonly ALL and AML.

ALL presenting with hypercalcaemia has a distinctive clinical profile, and most of the reported cases show certain common characteristics [6, 7, 8]. The children tend to be older (second decade of life) and have normal or low white blood cell (WBC) counts with rare or absent circulating blasts due to which their diagnosis is often delayed. The blasts characteristically show a pre-B ALL phenotype with aberrant expression of CD13 and other myeloid antigens and often are positive for t(17;19) on cytogenetic studies. Skeletal survey usually reveals disseminated osteolytic lesions and patients characteristically have chemorefractory disease. Our patient also had a low WBC count (2,300/mm3), a pre-B immune-phenotype with coexpression of CD13 and 33, and disseminated bone disease. He also had chemorefractory disease and received multiple lines of intensive multidrug chemotherapy [9, 10]. Subsequently, he went into remission only after the third line of induction chemotherapy. He had a HLA-matched sibling, and we planned for a allogenic stem cell transplant [11]. However, within one month of attaining remission, he relapsed and died.

Inukai *et al* [7] in their series of 22 patients with ALL and hypercalcaemia reported a lower incidence of severe anaemia (Hb < 8 g/dl) and thrombocytopenia (platelet count < 1 lakh/mm3) in this subgroup of patients compared to a historical cohort without hypercalcaemia.

### Mechanism of hypercalcaemia

The most common cause of malignancy-related hypercalcaemia is ectopic production of PTHrP. The cause in ALL is not well defined and may be related to the production of an osteoclast activating factor by leukaemic blasts. Other factors such as TGF-α, TNF, IL-1, PGE2 have also been implicated [6, 7, 12]. In one series of 21 patients [7], 11 had PTHrP-mediated hypercalcaemia, while two patients had raised TNF-α and IL-6.

### Management of hypercalcaemia

Severe hypercalcaemia as seen in our patient is life-threatening and can lead to cardiac arrhythmias, renal failure, and coma. Treatment consists of intense saline diuresis, frusemide, calcitonin, and bisphosphonates. The effect of bisphosphonates takes about 12 hours to commence, while calcitonin acts rapidly. However, the effect of calcitonin is short-lived and patients soon develop tolerance due to down-regulation of calcitonin receptors on osteoclasts. Steroids have an important adjunctive role as they not only reduce hypercalcaemia but also have an antileukaemic effect.

Our patient did not respond to diuresis, steroids, or calcitonin and required bisphosphonates which rapidly corrected the hypercalcaemia [13]. In the series by Inukai *et al* [7], 10 of 12 patients required i.v. bisphosphonate therapy. The authors also noted that renal dysfunction resolved faster in patients who received bisphosphonates, but most of these patients later needed prolonged parenteral calcium supplementation for hypocalcaemia. Our patient also required intravenous calcium and magnesium supplementation for nearly 3 weeks. Tumour lysis induced hypocalcaemia could have further compounded the metabolic derangement.

### Radiology

Radiological changes in acute leukaemia include osteopenia, lytic and sclerotic lesions, transverse metaphyseal bands, intra-medullary osteolytic mottling, and periosteal new bone formation. These changes have been reported in 41–70% of children with ALL [14]. However, destructive bone involvement with hypercalcaemia as observed in our patient is uncommon. The prognostic implication of bone lesions in ALL is unclear.

### Biology and outcome of the leukaemia

There are several case reports describing association of t(17;19) in children with ALL and hypercalcaemia [15]. t(17;19) is present in less than 1% of childhood ALL [16]. The t(17;19) (q22;p13) translocation generates the E2A-HLF chimeric transcription factor and is associated with a poor prognosis [17]. E2A-HLF can block apoptosis induced by the intrinsic mitochondrial pathway and has a central role in leukemogenesis and chemoresistance [18]. This has consistently been associated with hypercalcaemia, acquired coagulation abnormalities and DIC and chemorefractory disease. Other unique features in this subgroup include older age at onset, CD33 coexpression on leukaemic blasts, and L2 phenotype. Authors have suggested that this clinical profile should strongly suggest t(17;19). Our patient who had most of these features had normal cytogenetics on conventional karyotyping, but this translocation is best detected by fluorescent in situ hybridisation which was not done in our patient.

Hence, hypercalcaemia may itself not be a poor prognostic factor. Its strong association with t(17;19) may be responsible for the poor outcome reported in patients with hypercalcaemia. Our patient also required repeated cycles of chemotherapy before attaining remission for 1 month following which he relapsed and died.

### Conclusion

Hypercalcaemia at presentation in ALL, though rare, has important prognostic implications and can identify a subgroup of patients who are likely to be refractory to conventional chemotherapy. These patients usually have a distinctive clinical and cytogenetic profile and may be candidates for early intensification of treatment as response to conventional chemotherapeutic agents is poor.

## Figures and Tables

**Figure 1.**
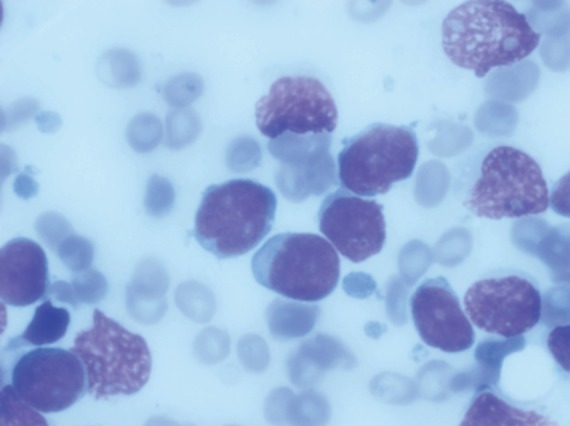
Bone marrow aspiration showing 59% lymphoblast.

**Figure 2.**
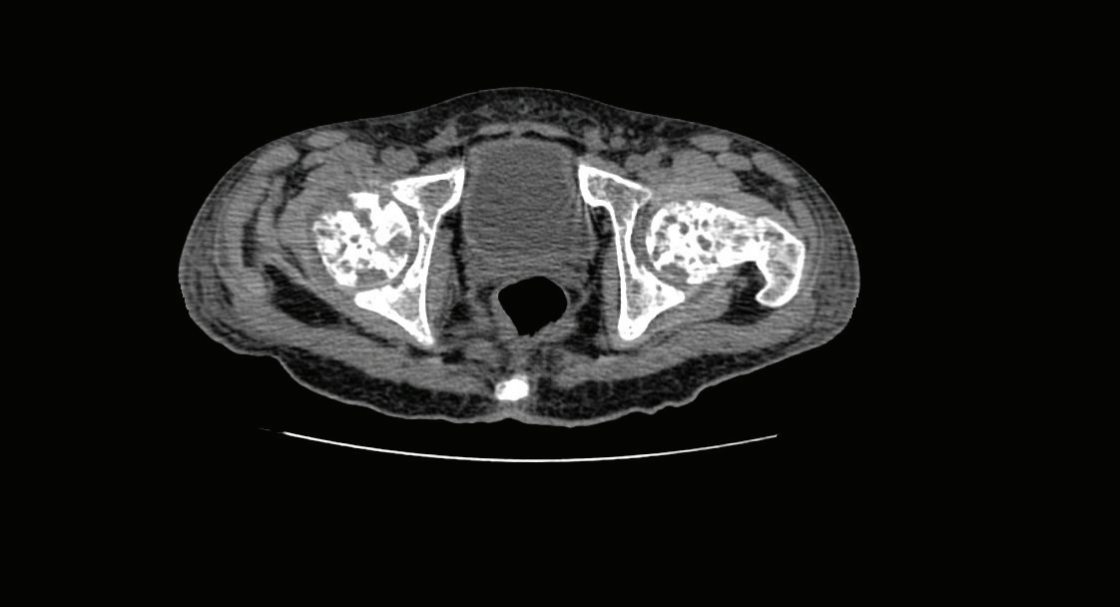
Computed tomography (CT) of the pelvis showed multiple lytic lesions.

**Figure 3.**
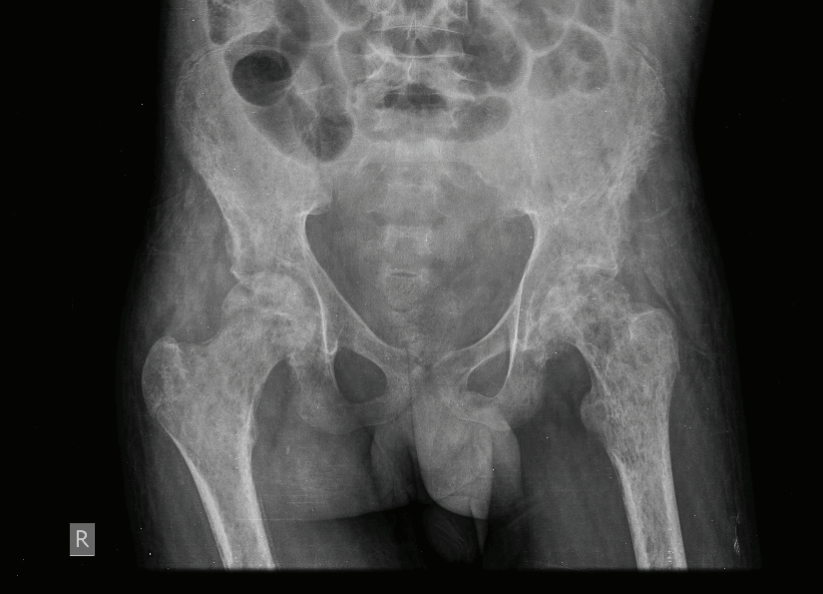
X-ray of the pelvis showed multiple permeative lesions involving the pelvic bones and the visualised proximal femurs.

**Figure 4.**
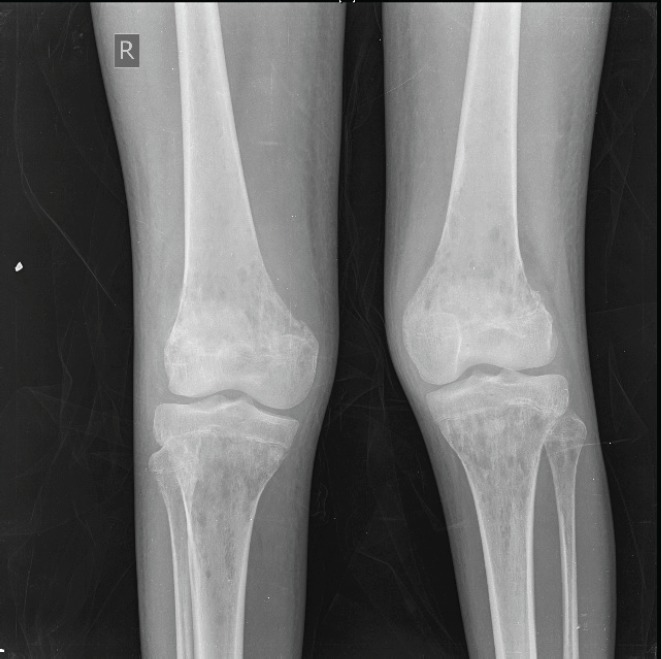
X-ray of the knee joint showing multiple lytic in the bones around the knee joint bilaterally.
